# A comprehensive analysis of G-protein-signaling modulator 2 as a prognostic and diagnostic marker for pan-cancer

**DOI:** 10.3389/fgene.2022.984714

**Published:** 2022-09-16

**Authors:** Lei-Ming Hu, Xue-Hai Ou, Shao-Yan Shi

**Affiliations:** Department of Hand Surgery, Honghui Hospital, Xi’an Jiaotong University, Xi’an, Shaanxi, China

**Keywords:** *Gpsm2*, prognosis, phosphorylation, gene mutation, immune infiltration, pan-cancer

## Abstract

**Background:** G-protein signaling modulator 2 (*GPSM2*) maintains cell polarization and regulates the cell cycle. Recent studies have shown that it is highly expressed in various tumors, but its pan-cancer analysis has not been reported.

**Methods:** First, we analyzed the differential *GPSM2* expression in normal and cancer tissues by the Cancer Genome Atlas (TCGA), Genotype-Tissue Expression (GTEx) and Human Protein Atlas databases and investigated its expression effect on the survival of cancer patients by gene expression profiling interactive analysis 2 (GEPIA2). Second, we analyzed the *GPSM2* phosphorylation level using the clinical proteomic tumor analysis consortium dataset. In addition, we investigated *GPSM2* gene mutations in human tumor specimens and the impact of gene mutations on patient survival. Finally, we analyzed the relationship between *GPSM2* expression and cellular immune infiltration through the TIMER 2.0 database. Meanwhile, the possible signaling pathway of the gene was analyzed by the Gene Ontology (GO)| Kyoto Encyclopedia of Genes and Genomes (KEGG) pathway to explore its potential mechanism.

**Results:** *GPSM2* is overexpressed in most cancers, which leads to reduced overall survival (OS) and disease-free survival in patients. The results of phosphorylation analysis suggest that tumor development involves a complex *GPSM2* phosphorylation process. We identified *GPSM2* mutation loci with the highest frequency of mutations in uterine corpus endometrial carcinoma (UCEC), and this mutation increased progression-free survival and overall survival in uterine corpus endometrial carcinoma patients. Finally, we found that the role of *GPSM2* in tumors may be associated with cellular immune infiltration. Gene Ontology|KEGG pathway analysis showed that the enrichment pathways were mainly “mitotic nuclear division,” “chromosome segregation,” and “spindle.”

**Conclusions:** Our pan-cancer analysis provides a comprehensive overview of the oncogenic roles and potential mechanisms of *GPSM2* in multiple human cancers.

## Introduction

G-protein signaling modulator 2 (*GPSM2*)/Leu-Gly-Asn repeat-enriched protein (LGN), which regulates the activation of G proteins, receives extracellular signals and causes cellular responses (Blumer et al., 2006). *GPSM2* is necessary to orient the mitotic spindle during cell division and is essential in maintaining cell polarity and participating in cell cycle regulation (Du et al., 2001; Woodard et al., 2010). It contains 10 copies of an LGN repeat in the N‐terminal portion and 4 GoLoco motifs in the C‐terminal part of the protein and is widely expressed in human tissues (Mochizuki et al., 1996). In addition, *GPSM2* gene deletion or mutation is likely to cause defects in cell polarity, resulting in characteristic brain malformations and nonsyndromic hearing loss (Doherty et al., 2012).

In addition to being expressed in normal human tissues, *GPSM2* is also involved in disease processes. Recent work identified aberrant *GPSM2* expressions in various tumors, such as liver hepatocellular carcinoma (LIHC) (He et al., 2017) and pancreatic cancer (Dang et al., 2019). Meanwhile, many studies have reported that *GPSM2* can be identified as a prognostic factor in LIHC that promotes tumor proliferation and metastasis (Yang et al., 2020). However, the role of *GPSM2* in tumors and the specific mechanisms remain uncertain.

To explore the *GPSM2* expression profile in pan-cancer analysis, we used a dataset from The Cancer Genome Atlas (TCGA) database. We compared *GPSM2* expression in various tumors and considered survival status, protein phosphorylation, gene alteration, immune cell infiltration and related cellular pathways. This comprehensive analysis helps reveal the *GPSM2* mechanism in human tumors, which is also helpful in predicting tumor prognosis and providing implications for targeted cancer therapy.

## Materials and methods

### Analysis of G-protein signaling modulator 2 expression in normal and tumor tissues

The TIMER 2.0 database (http://timer.cistrome.org/) was used to analyze *GPSM2* expression between different tumors and adjacent normal tissues. Then, we used gene expression profiling interactive analysis 2 (GEPIA2) (http://gepia2.cancer-pku.cn/#analysis) to acquire box plots of the genotype-tissue expression (GTEx) database. Setting *p* value cutoff = 0.01, log_2_ fold change (FC) cutoff = 1, and “matching TCGA normal and GTEx data.” Then, GEPIA 2 was used to analyze the *GPSM2* protein level in different cancers. Finally, *GPSM2* expression at different pathological stages of various cancers was analyzed by GEPIA2. We used log_2_ [transcripts per million (TPM)+1)] for log-scale to obtain expression data to produce violin plots.

### Immunohistochemical staining

To evaluate the difference in *GPSM2* expression, we performed an analysis by TCGA + GTEx dataset and selected cancer types with high *GPSM2* expression in tumors; downloaded from the Human Protein Atlas (HPA) (https://www.proteinatlas.org/) for *GPSM2* expression IHC images in normal and seven tumor tissues, including LIHC, kidney renal clear cell carcinoma (KIRC), breast cancer (BRCA), colon adenocarcinoma (COAD), prostate adenocarcinoma (PRAD), stomach adenocarcinoma (STAD), and ovarian serous cystadenocarcinoma (OV), were downloaded from the HPA database and analyzed.

### Western bolt

The tissues were fully lysed using RIPA lysis slow at 4°C; the supernatant was collected by centrifugation at 4°C at 12,000 rpm for 15 min, and the protein concentration was measured according to the Bradford method. Then, 4X protein loading buffer was added, heated by boiling in a water bath for 10 min, and stored at −80°C. Proteins (20–40 μg) were electrophoresed on sodium dodecyl sulfate-polyacrylamide gel electrophoresis (SDS-PAGE) gels at different concentrations (8%, 10% or 12%, depending on protein molecular weight) and then transferred to polyvinylidene difluoride (PVDF) membranes for transmembrane processing. The membranes were closed in the solution for 1 h and then incubated with the corresponding primary antibody overnight at 4°C. Primary antibodies included *GPSM2* (1:100) and β-actin (1:1,000). The membranes were washed three times for 10 min each in TBST solution the following day. The membranes were incubated with the corresponding secondary antibodies for 2 h at 4°C, and the TBST solution was washed three times for 10 min each. Finally, color development was performed using ECL luminescent solution exposed on a gel imaging system and images were acquired. Grayscale values of protein bands were analyzed using ImageJ software.

### Survival prognosis analysis

We used GEPIA2 to obtain the overall survival (OS) and disease-free survival (DFS) significance map data and *GPSM2* survival plots. The most differentially expressed cancers were selected for survival analysis, and cutoff-high (50%) and cutoff-low (50%) values were used as the expression thresholds for splitting the high- and low-expression cohorts (Tang et al., 2019). The hazard ratio was calculated based on the Cox PH model, and the 95% confidence interval was set as the selection of outcome criteria for survival curve plotting. The log-rank test was used in the hypothesis testing. The threshold was set as a Cox *p* value <0.05.

### Prognostic analysis and clinical model prediction of G-protein signaling modulator 2 in liver hepatocellular carcinoma

By analyzing the significant degree of *GPSM2* expression across cancers, we selected LIHC further to explore the impact of *GPSM2* expression on cancer prognosis. Multi-factor Cox regression analysis of LIHC was performed using the R package (version 3.6.3), and factors influencing *p < 0.05* were statistically analyzed using the rms R package. To personalize the prognosis of patients with LIHC, KM plots of *GPSM2* on LIHC, nomogram plots of clinical characteristics and calibration plots were drawn (Liu et al., 2018).

### Genetic alteration analysis

Genetic alteration analysis of *GPSM2* in TCGA pan-cancer was performed by using cBio Cancer Genomics Portal (cBioPortal) (https://www.cbioportal.org/), which maps the three-dimensional structure of alteration frequency, mutation type, mutation site, copy number alteration (CNA) and protein structure. Then, the effect of *GPSM2* mutations on survival was analyzed in the uterine corpus endometrial carcinoma (UCEC) single dataset, i.e., the comparison/survival module was selected in the TCGA-UCEC dataset and their OS, disease-specific survival (DSS), progression-free survival (PFS), and DFS survival curves.

### Phosphorylation analysis

We extracted the *GPSM2* phosphorylation data in normal and tumor tissues from the clinical proteomic tumor analysis consortium (CPTAC) dataset, annotated phosphorylation sites and plotted the corresponding box plots.

### Immunoinfiltration analysis

The TIMER database analyzed the relationship between *GPSM2* expression and immune infiltration in all tumors. We selected cancer-associated fibroblasts, neutrophils and endothelial cells for study. The EPIC, MCP-counter, TIDE, XCELL, CIBERSORT, CIBERSORT-ABS, QUANTISEQ and TIMER algorithms were used for immune infiltration assessment criteria to plot a heatmap of the correlation between *GPSM2* expression and immune infiltration.

### G-protein signaling modulator 2-related gene enrichment analysis

First, we used the STRING website (https://string-db.org/) for subsequent analysis of the protein-protein interaction (PPI) network. The following main parameters are set: the minimum interaction score required [“low confidence (0.150)”], the edge of the network meaning (“evidence”), the maximum number of interactors to be shown (“no more than 50 interactors” in the first shell) and the source of the active interaction (“experiments”).

The resulting PPI maps were then produced using Cytoscape version 3.9.1 software. The top 41 most relevant ranked genes for *GPSM2* were screened by 12 algorithms, such as betweenness, bottleneck, closeness, and degree, in the cytoHubba program. Then, the GEPIA2 tool was used to screen the top 100 genes with the highest correlation to *GPSM2* in all TCGA tumors.

Second, we performed intergenic Pearson correlation analysis between *GPSM2* and the selected genes. Mark the *p* values and correlation coefficients (R), display them in the corresponding plot positions and plot the correlation heat map.

Finally, we used the Gene Ontology (GO) | Kyoto Encyclopedia of Genes and Genomes (KEGG) for functional enrichment analysis. We used the clusterProfiler (Yu et al., 2012) package for enrichment analysis and the ggplot2 package for visualization.

## Results

### G-protein signaling modulator 2 expression analysis data

**FIGURE 1:**
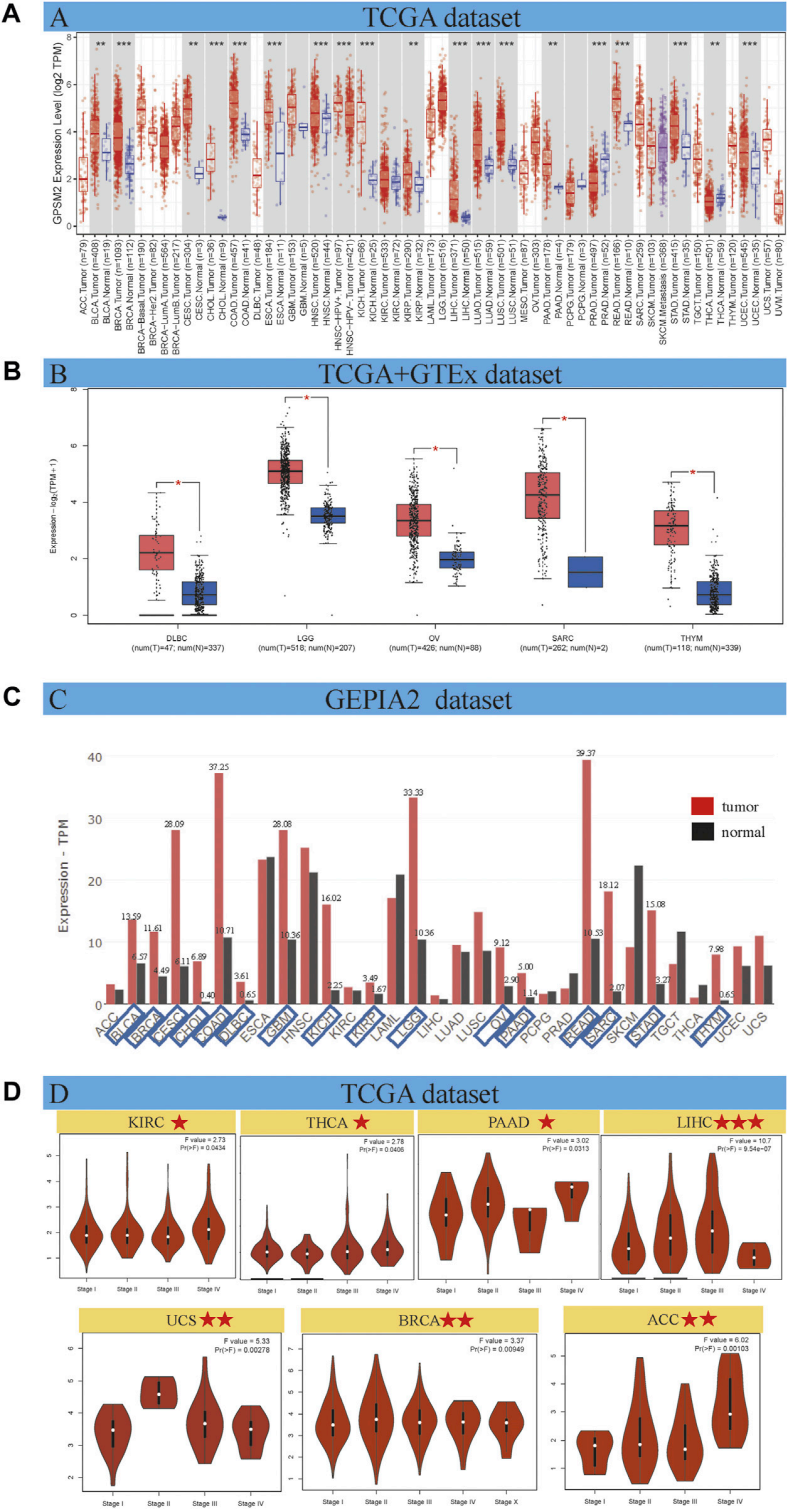
*GPSM2* expression in various tumors and pathological stages. **(A)** *GPSM2* expression difference between tumor and adjacent normal tissues. **(B)** The GTEx dataset shows a block diagram of the TCGA dataset lacking *GPSM2* expression data. **(C)** Differences in *GPSM2* total protein levels between cancer and normal tissues. **(D)** Stage-specific *GPSM2* expression across cancers (*p < 0.05; **p < 0.01; ***p < 0.001).

As shown in Figure 1A, the expression differences were divided into four categories: 1) *GPSM2* expression in cancer tissues was higher than in adjacent normal tissues. Among them, the differences between bladder urothelial carcinoma (BLCA), BRCA, cholangiocarcinoma (CHOL), COAD, esophageal carcinoma (ESCA), head and neck squamous cell carcinoma (HNSC), kidney chromophobe (KICH), LIHC, lung adenocarcinoma (LUAD), lung squamous cell carcinoma (LUSC), rectum adenocarcinoma (READ), STAD and UCEC were the largest (*p* < 0.001). In addition, BLCA, ceramic square cell carcinoma (CESC), kidney renal papillary cell carcinoma (KIRP), pancreatic adenocarcinoma (PAAD) and thyroid carcinoma (THCA) were higher than those in normal tissues (*p* < 0.01). 2) A few tumors, such as glioblastoma multiform (GBM), KIRC, pheochromocytoma and paraganglioma (PCPG), and skin cutaneous melanoma (SKCM), have no differential expression with normal tissues. 3) *GPSM2* expression in PRAD was significantly lower than that in normal tissues (*p* < 0.001). 4) The TCGA dataset cannot reflect the expression of all cancers. In adrenocortical carcinoma (ACC), lymphoid neoplasm diffuse large B-cell lymphoma (DLBC), acute myeloid leukemia (LAML), brain lower grade glioma (LGG), mesothelioma (MESO), OV, sarcoma (SARC), testicular germ cell tumors (TGCT), thymic carcinoma (THYM), uterine carcinosarcoma (UCS) and uveal melanoma (UVM) did not differ from normal tissues.

The GTEx dataset improves the expression difference data lacking in the above TCGA dataset. We found that *GPSM2* was highly expressed in DLBC, LGG, OV, SARC and THYM tumor tissues (Figure 1B, *p* < 0.05). We found no significant differences in ACC, LAML, MESO, TGCT, UCS, or UVM. In general, the expression level of *GPSM2* in most human tumors is higher than in normal tissues.

Gene expression is ultimately reflected at the protein level. Therefore, we used the GEPIA2 dataset to evaluate the total protein content of *GPSM2* across cancers. We know that the *GPSM2* protein level in most cancers is higher than in corresponding normal tissues. We labeled tumor types whose total protein content was more than twice that of normal tissues. CHOL, DLBC, KICH, SARA and THYM had higher protein contents (Figure 1C).

On the other hand, GEPIA 2 was used to analyze the correlation between *GPSM2* expression in different pathological stages of tumors and found stage-specific changes in KIRC, THCA, PAAD, LIHC, UCS, BRCA and ACC (Figure 1D, *p* < 0.05).

### Immunohistochemistry of G-protein signaling modulator 2 in tumor and normal tissues

**FIGURE 2:**
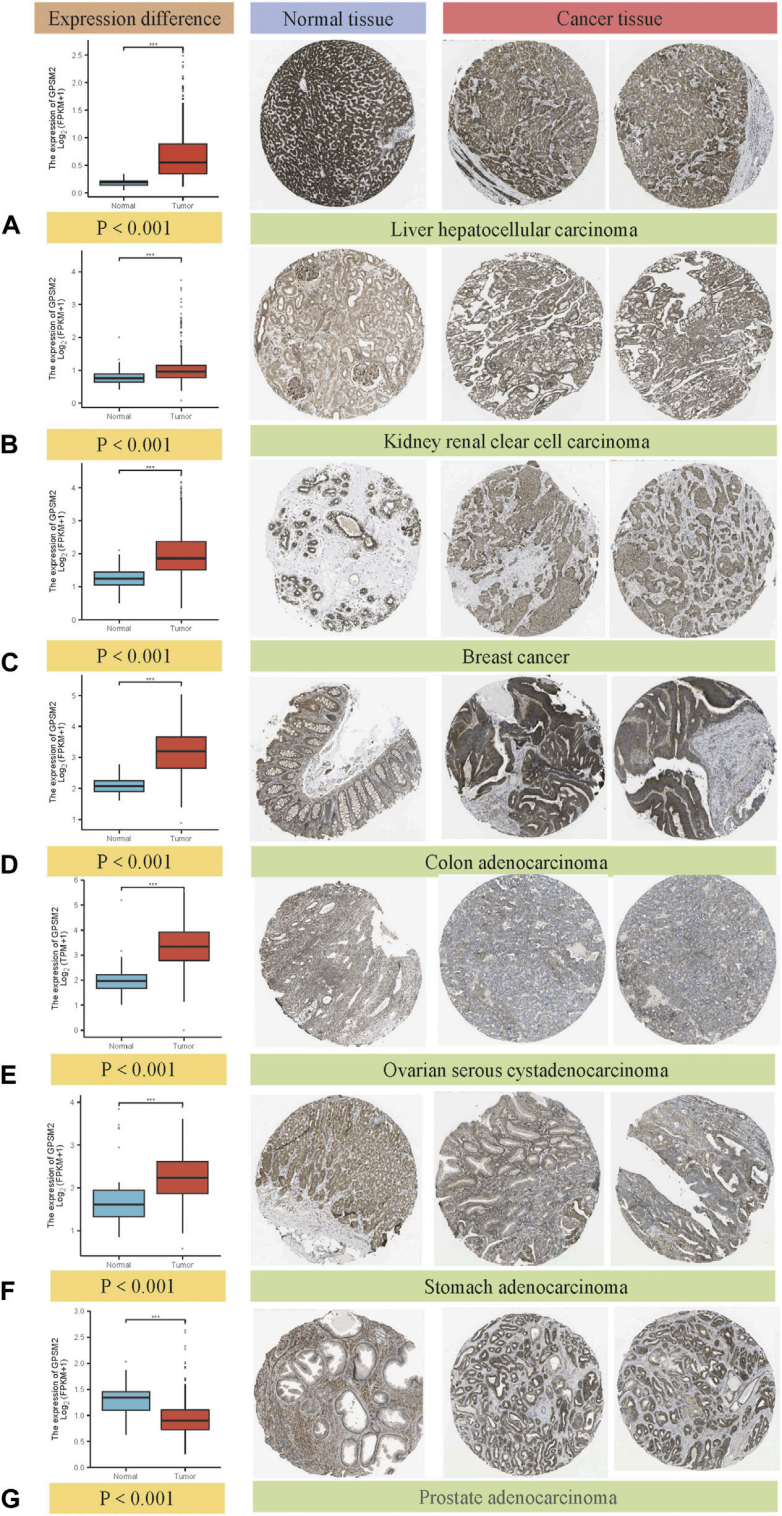
Box diagram of *GPSM2* expression between normal and tumor tissues (left), comparison of immunohistochemical staining between normal (middle) and tumor tissues (right). *GPSM2* expression in **(A)** LIHC, **(B)** KIRC, **(C)** BRCA, **(D)** COAD, **(E)** OV and **(F)** STAD was significantly increased, and the expression in **(G)** PRAD was lower than that in normal tissues.

Comparing the immunohistochemical results provided by the HPA dataset in the TCGA dataset, we selected seven types that had the most apparent difference between tumor tissues and normal tissues. *GPSM2* expression in LIHC, KIRC, BRCA, COAD, OV and STAD was significantly increased, but *GPSM2* expression in PRAD was lower than that in normal tissues (Figures 2A–G) (we selected immunohistochemical images with >75% tumor cells and moderate or vigorous staining).

### Expression of G-protein signaling modulator 2 in liver hepatocellular carcinoma and normal tissues

**FIGURE 3:**
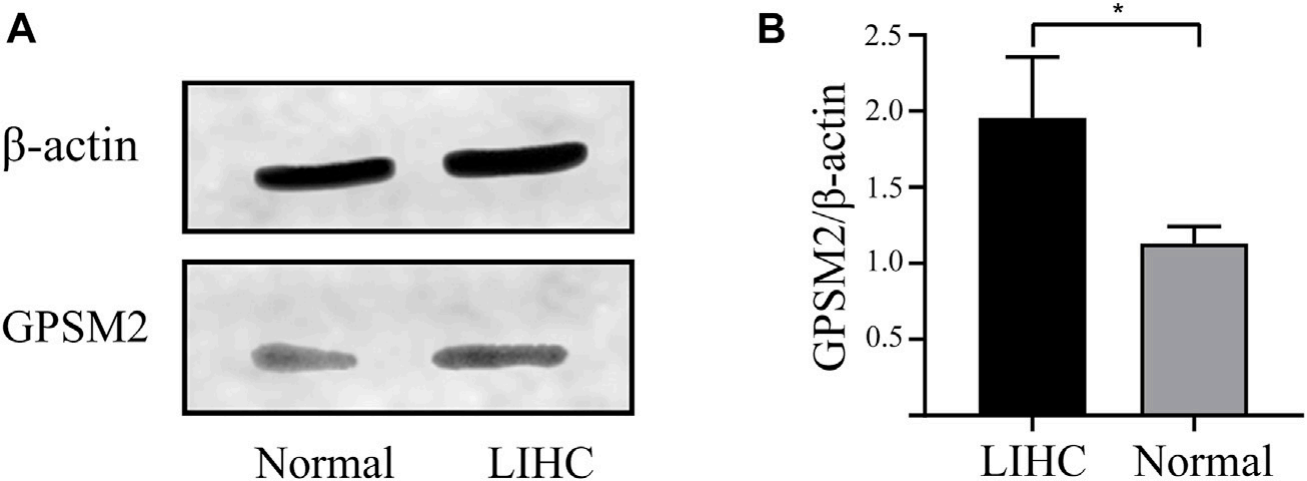
Expression of *GPSM2* in human normal and LIHC tissues. **(A)** Protein bands of *GPSM2* expression in normal and LIHC tissues. **(B)** Histogram of *GPSM2* expression in normal and LIHC tissues.

To explore the importance of *GPSM2* in tumors, we extracted total proteins from normal liver and LIHC tissues in humans and verified their protein expression by Western blot. The *GPSM2* expression was significantly higher than that of normal tissues (Figures 3A,B), suggesting a predictive role for *GPSM2* in tumors.

### Survival analysis results

**FIGURE 4:**
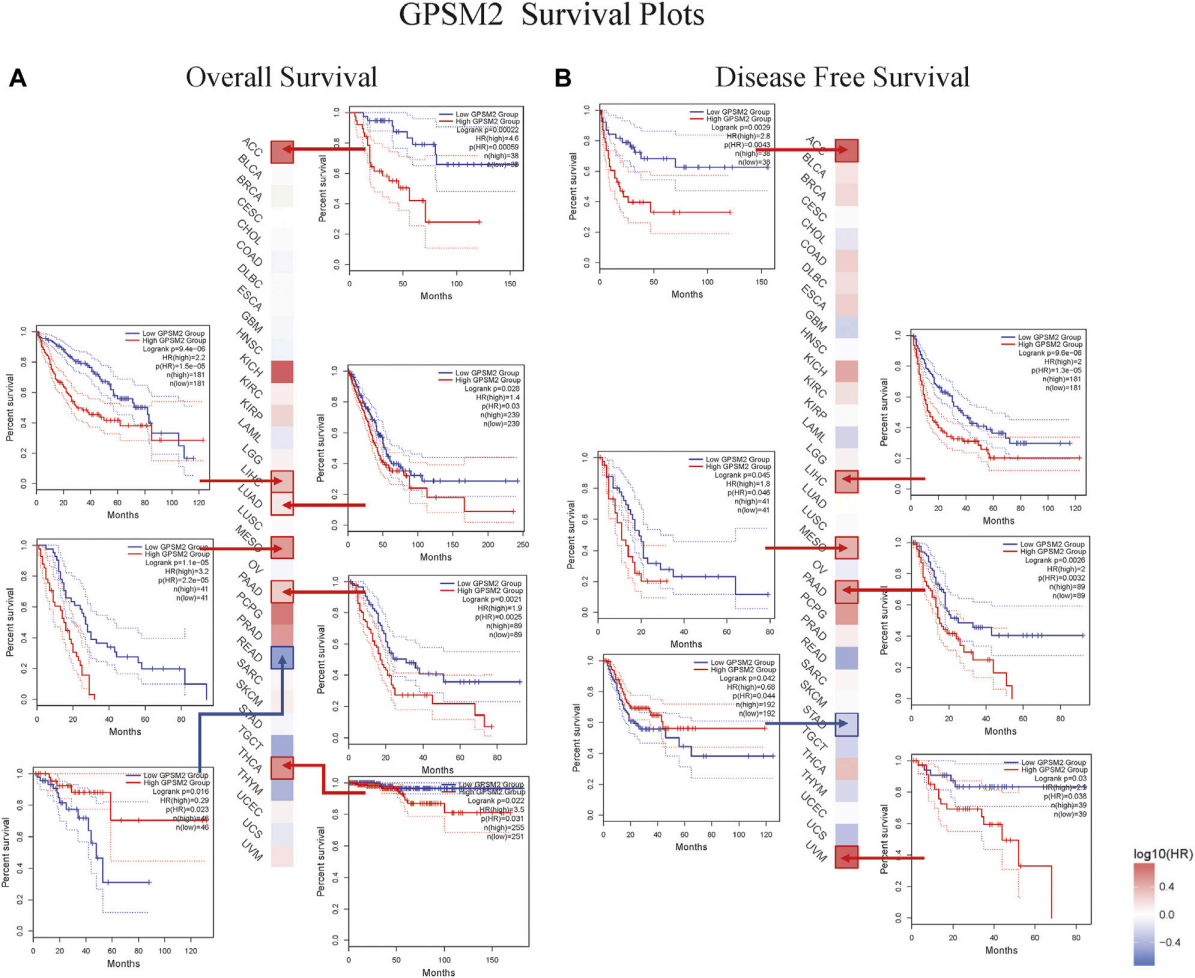
Prognostic survival map and Kaplan–Meier curve of *GPSM2* expression in TCGA pan-cancer patients. **(A)** The relationship between *GPSM2* expression and OS. **(B)** The relationship between *GPSM2* expression and DFS.

This chapter focused on the relationship between *GPSM2* expression and prognosis. First, we divided the patients into two groups according to *GPSM2* expression on the survival map and then studied the correlation between *GPSM2* expression and patient prognosis. In terms of OS, high *GPSM2* expression was associated with poor OS prognosis in ACC (*p* = 5.9e-04), LIHC (*p* = 1.5e-05), LUAD (*P* = 3e-02), PAAD (*p* = 2.5e-03), MESO (*p* = 2.2e-05), and THCA (*p* = 3.1e-02) (Figure 4A). In terms of DFS, high *GPSM2* expression was associated with poor prognosis in ACC (*p* = 4.3e-03), LIHC (*p* = 1.3e-05), MESO (*p* = 4.6e-02), PAAD (*p* = 3.2e-03), and UVM (*p* = 3.8e-02) (Figure 4B).

### Prognostic analysis and clinical predictive model of G-protein signaling modulator 2 in liver hepatocellular carcinoma

**FIGURE 5:**
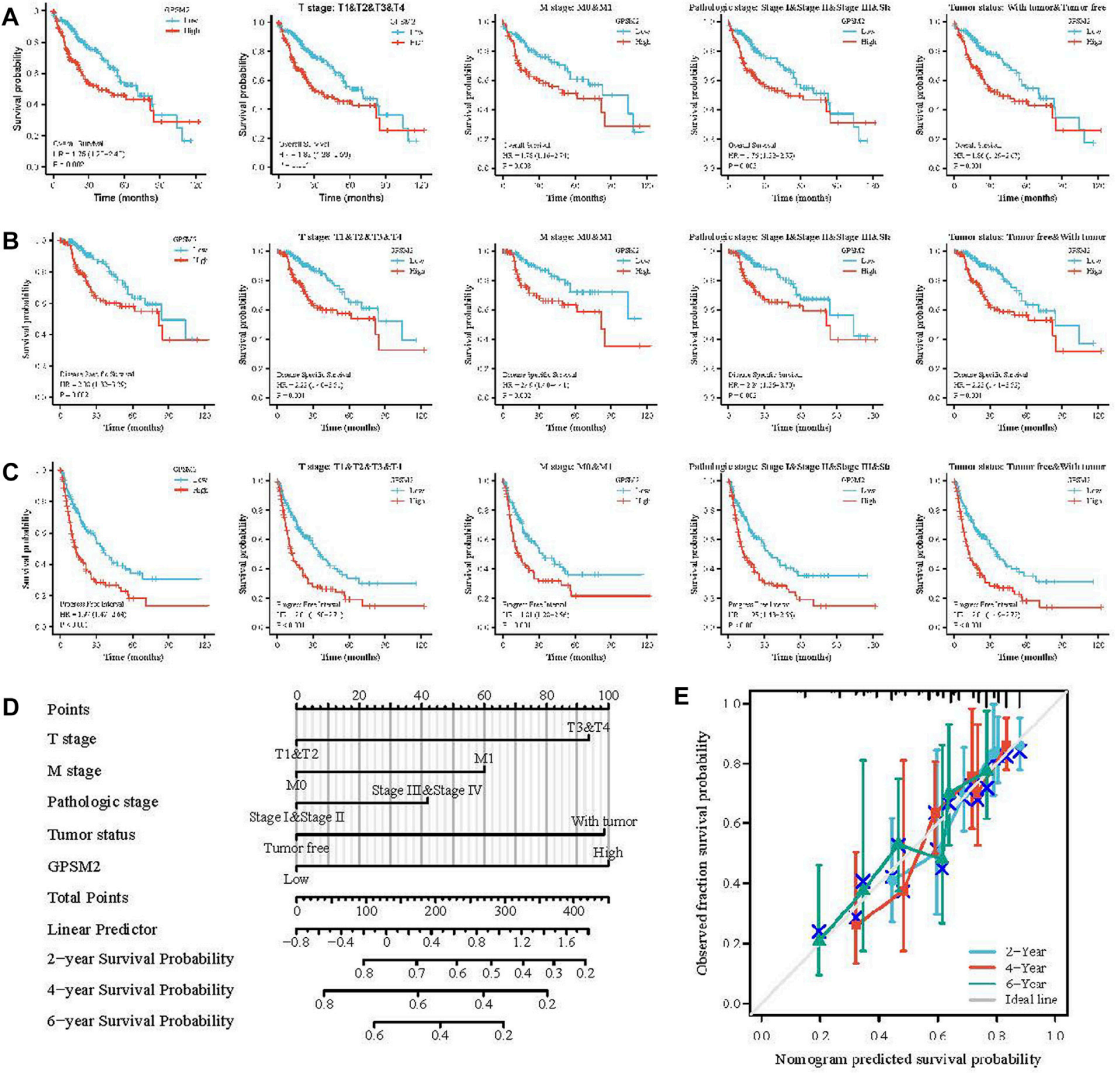
Prognosis of *GPSM2* in LIHC tumors and *GPSM2* prediction model in LIHC patients. **(A–C)** Correlation between *GPSM2* and OS, DSS, and PFI in different clinical subgroups of LIHC. **(D)** Colinear plots of prognostic predictors and annual survival in LIHC. **(E)** Colinear calibration curves.

High *GPSM2* expression was associated with reduced OS, DSS and PFI, including OS [HR = 1.75, *p* = 0.002] (Figure 5A), DSS (HR = 2.08, *p* = 0.002) (Figure 5B) and PFI (HR = 1.97, *p* < 0.001) (Figure 5C). In addition, we investigated the correlation between *GPSM2* expression and prognosis in different clinical subgroups (T stage, M stage, pathological stage, tumor status) of LIHC. OS included T stage (*p* = 0.001), M stage (*p* = 0.008), pathological stage (*p* = 0.003), and tumor status (*p* = 0.001) (Figure 5A); DSS included T stage (*p* = 0.001), M stage (*p* = 0.002), pathological stage (*p* = 0.002), and tumor status (*p* = 0.001) (Figure 5B); and PFI included stage (*p* < 0.001), M stage (*p* = 0.001), pathological stage (*p* < 0.001), and tumor status (*p* < 0.001) (Figure 5C).

Finally, nomogram plots were constructed to predict the 2-, 4-, and 6-year survival rates of LIHC patients. Five prognostic factors, T stage, M stage, pathologic stage, tumor status, and *GPSM2* expression, were included in the model. The yearly prognostic survival probabilities of patients were obtained in the lower graphs after calculating the total scores of each variable for LIHC patients using the point scale (Figure 5D), and the results of the calibration curve prediction in the nomogram plots were found to be generally consistent with the patients’ observations (Figure 5E).

### Protein phosphorylation analysis

**FIGURE 6:**
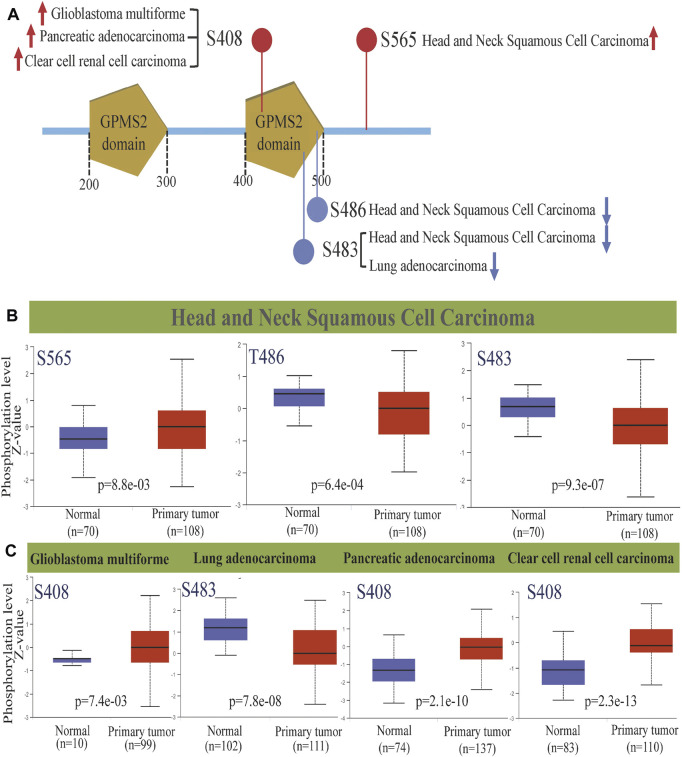
*GPSM2* protein phosphorylation diagram across cancers. **(A)** *GPSM2* protein phosphorylation sites were detected. **(B)** Box diagram of *GPSM2*-related protein phosphorylation levels in HNSC. **(C)** Box diagram of *GPSM2*-related protein phosphorylation levels in GLMU, LUAD, PAAD, and KIRC.

Protein phosphorylation, the process by which the phosphate group of ATP is transferred to amino acid residues of substrate proteins by the action of protein kinases, is the most fundamental, pervasive and essential mechanism for regulating and controlling protein activity and function. It is also a key marker of tumorigenesis, development, evolution and targeted therapy (Meyer et al., 2021). Therefore, we analyzed the *GPSM2* phosphorylation degree between normal and tumor tissues by the CPTAC dataset and screened five tumor tissues with meaningful differences (Figure 6A). Among them, the *GPSM2* phosphorylation level at the S408 site in glioblastoma multiforme (GLMU) PAADand KIRC and the S565 site in HNSC were significantly increased (Figure 6B, Figure 6C). In contrast, the *GPSM2* phosphorylation levels at the T486 and S483 sites in HNSC and the S483 site in LUAD were lower than those in normal tissues (Figures 6B,C).

### Mutation status of G-protein signaling modulator 2

**FIGURE 7:**
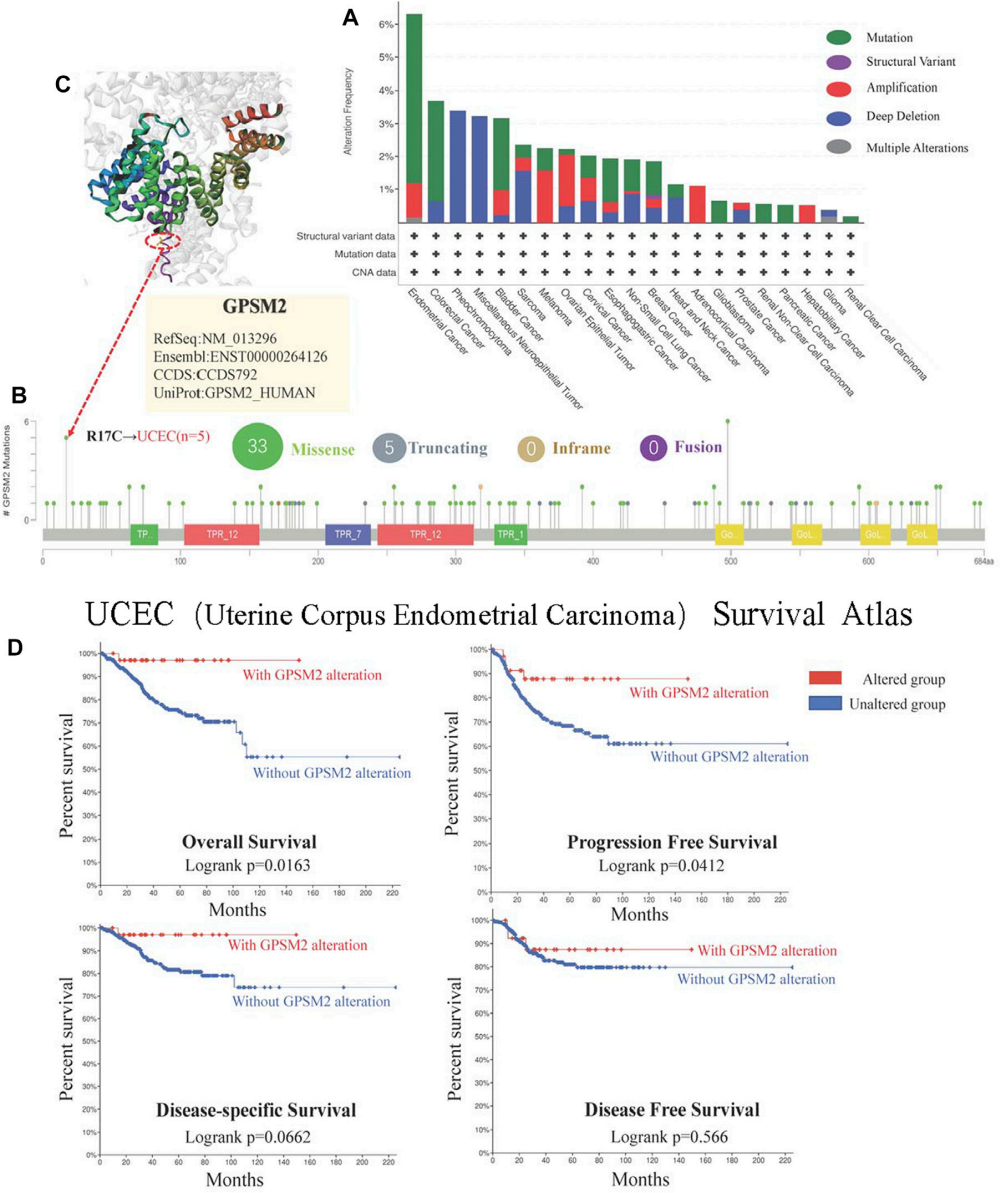
Mutations of *GPSM2* in TCGA pan-cancer. **(A)** The mutation type and frequency of *GPSM2* in tumors; **(B)** The change frequency of the *GPSM2* gene structure and its mutation sites; **(C)** The position of the most frequent mutation site (R17C) in the 3D structure of the *GPSM2* protein. **(D)** Correlation between UCEC with *GPSM2* mutation and OS, DSS, DFS, and PFS.

In the long run, this small probability event can lead to the occurrence and evolution of cancer. Therefore, studying *GPSM2* gene changes in human tumor samples will help us clarify tumor pathogenesis and select therapeutic targets. We found that the tumors with the highest *GPSM2* “Mutation” frequency (>6%) were UCEC. The highest incidence of “amplification” CNA was ACC (>4%) (Figure 7A). As shown in Figure 7B, the mutation sites of the *GPSM2* gene are mapped. However, no dominant genetic mutation type was found, and the R17C mutation was detected in 5 cases of UCEC. To visualize the mutation location of the R17C site, we mapped the 3D structure of the *GPSM2* protein and located and marked R17C (Figure 7C). In addition, we used the “cBioPortal” tool to explore the relationship between UCEC and the prognosis of clinical patients. The results showed that patients with *GPSM2* mutations had a better prognosis in terms of PFS (*p* = 0.0412) and OS (*p* = 0.0163), but there was no significant difference in DFS (*p* = 0.566) or DSS (*p* = 0.0662) (Figure 7D).

### Immune infiltration analysis results

**FIGURE 8:**
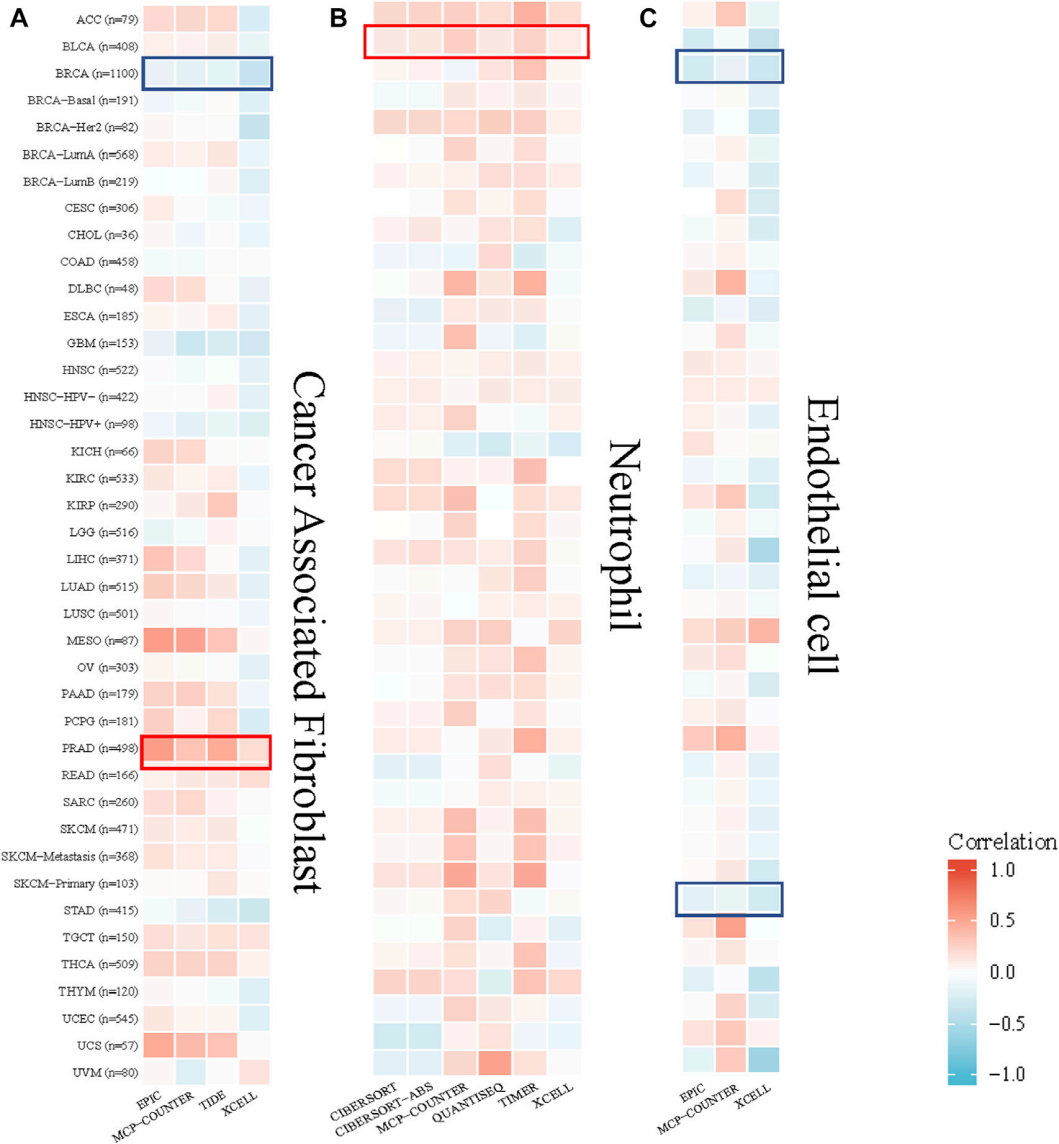
Correlation between *GPSM2* expression and cancer-associated fibroblast, neutrophil and endothelial cell infiltration. **(A–C)** Heatmap of the correlation between the infiltration levels of cancer-associated fibroblasts, neutrophils and endothelial cells and *GPSM2* expression.

The TIMER algorithm was used to explore the correlation between the cancer-associated fibroblast, neutrophil, endothelial cell infiltration level and *GPSM2* expression in TCGA pan-cancer. The results showed that *GPSM2* expression was positively correlated with the estimated cancer-associated fibroblast infiltration value in PRAD and negatively correlated with BRCA. There was a positive correlation between *GPSM2* expression and neutrophils in BLCA. In addition, *GPSM2* expression in BRCA and STAD was negatively associated with endothelial cell infiltration (Figure 8).

### G-protein signaling modulator 2 similar gene enrichment analysis

**FIGURE 9:**
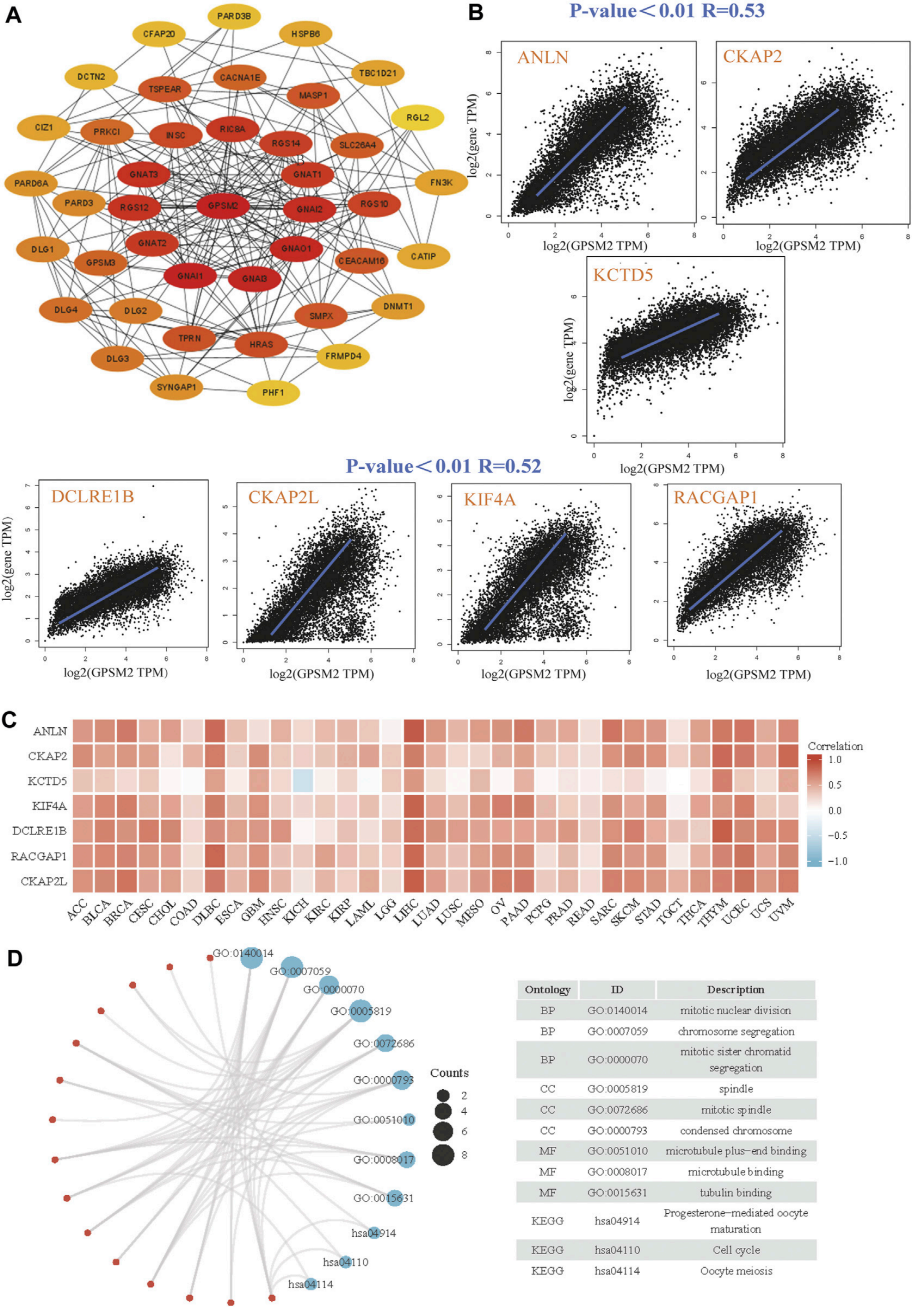
Enrichment and pathway analysis of *GPSM2*-related genes. **(A)** Known *GPSM2* binding-protein string network. **(B)** The GEPIA2 dataset shows the expression correlation between *GPSM2* and representative genes (ANLN, CKAP2, KCTD5, DCLRE1B, CKAP2 L, KIF4A, and RACGAP1) of the top *GPSM2*-correlated genes. **(C)** In TCGA pan-cancer, *GPSM2* expression and ANLN, CKAP2, KCTD5, DCLRE1B, CKAP2 L, KIF4A, and RACGAP1 were correlated with the heatmap. **(D)** GO|KEGG pathway analysis based on *GPSM2* and its interacting genes.

Finally, we screened *GPSM2*-interacting proteins and *GPSM2*-related genes in the GEPIA dataset for pathway enrichment analysis. The first 41 species that interacted most closely with the *GPSM2* protein were selected by STRING and Cytoscape tools (Figure 9A). Then, through the *GPSM2* expression data in GEPIA2+TCGA pan-cancer, the top 100 genes with the strongest correlation with *GPSM2* expression were screened. Among them, *GPSM2* expression was positively correlated with anillin (ANLN), cytoskeleton-associated protein 2 (CKAP2), potassium channel tetramerization domain-5 (KCTD5), DNA cross-link repair 1B (DCLRE1B), CKAP2 like (CKAP2 L), kinesin family member 4A (KIF4A), and RAC GTPase activating protein 1 (RACGAP1) (Figure 9B). Heatmap data showed that *GPSM2* had a strong positive correlation with the seven genes above (Figure 9C). We combined the two datasets to perform GO and KEGG enrichment analyses. The results revealed that the main pathways were “mitotic nuclear division,” “chromosome segregation” and “spindle” (Figure 9D).

## Discussion

Cancer has long been a worldwide clinical challenge, resulting in at least tens of millions of deaths each year. Although current treatments such as surgery, radiation therapy and medication are usually effective, they can also impose a significant financial burden and physical toll on patients. A better understanding of the molecular basis of cancer and the emergence of new diagnostic techniques will help eliminate cancer cells and improve cancer treatment. Therefore, it is clear that studying gene expression and epigenetic changes in cancer cells and the underlying pathogenesis is beneficial for early detection and diagnosis. At the same time, it appears crucial for medical professionals to intervene in treatment by using minimally invasive routes that are relatively less damaging (Zaimy et al., 2017).

The development of molecularly targeted anticancer drugs has improved clinical outcomes for many cancer patients, but the number of patients benefiting from them is relatively small. Therefore, there is an urgent need for further rapid development of new gene-targeted drugs. We analyzed the differences in *GPSM2* mRNA and protein expression levels by bioinformatics. We found that the transcript and protein levels of *GPSM2* were increased in most tumors compared to normal tissues (e.g., OV and THYM), suggesting a pro-cancer role for *GPSM2* in most tumors. Meanwhile, we verified that the expression of *GPSM2* in LIHC was higher than that in normal liver tissue by Western blot assay. In addition, *GPSM2* expression differed significantly between pathological stages and appeared to be upregulated at higher pathological stages. Dang et al. (Dang et al., 2021a) reported that all *GPSM* family members were significantly differentially expressed in BRCA, and their expression levels were also correlated with advanced tumor stage. At the same time, they found that higher *GPSM2* expression was associated with decreased survival in BRCA patients (Dang et al., 2021a).

Nevertheless, the expression and function of *GPSM2* depend on different tumor types. For example, Deng et al. (Deng et al., 2020a) found that *GPSM2* was downregulated in non-small cell lung cancer tissues, and knockdown of *GPSM2* promoted non-small cell cancer cell metastasis *in vitro* and *in vivo* and accelerated the epithelial-mesenchymal transition (EMT) process. Meanwhile, some scholars found that silencing *GPSM2* induced cell metastasis and EMT through the ERK/glycogen synthase kinase-3β/Snail pathway. Loss of *GPSM2* accelerates LUAD cell proliferation through the EGFR pathway (Deng et al., 2020b). This seems inconsistent with our findings, and we speculate that this is related to individual differences resulting in different genetic samples. Nevertheless, either result needs to be validated with further expanded clinical sample sizes.

In addition, we found that *GPSM2* overexpression was associated with poor prognosis in cancer patients (e.g., ACC and LIHC). In addition, *GPSM2* was associated with chronic pancreatitis, T stage, TNM stage and tumor grade, presumably as an independent prognostic factor (Zhou et al., 2021). Considering the small number of identified oncogenes and poor prognosis genes, this is a supplement to poor prognosis in cancer patients in terms of genes. We demonstrated the clinical predictive role of *GPSM2* in LIHC by drawing a nomogram, which showed that *GPSM2* could be an independent risk factor for LIHC, that high *GPSM2* expression is associated with poor prognosis in patients with LIHC, and that the calibration plot showed increased confidence in the predictive role of *GPSM2*.

Compared to normal cells, epigenetic alterations (altered gene expression without any alteration in the primary DNA sequence) are significant in tumor cells. Our results showed that the *GPSM2* phosphorylation level at the S408 site in GLMU, PAAD, KIRC and the S565 site in HNSC was significantly increased, but at the T486 and S483 sites in HNSC and the S483 site in LUAD, it was lower. Since no *GPSM2* phosphorylation site has been reported to be associated with cancer, we may be the first to report a phosphorylation site. We found that *GPSM2* phosphorylation was higher in some tumors, consistent with previous reports. For example, Fukukawa et al. (Fukukawa et al., 2010) confirmed the *GPSM2* upregulation in BRCA by semiquantitative RT-PCR and western-blot analysis, with the highest expression and phosphorylated form of *GPSM2* protein in the G2/M phase during the mitotic phase. Treatment with small interfering RNA targeting *GPSM2* resulted in incomplete cytokinesis and BRCA cells’ significant growth inhibition. Suggesting a vital role for *GPSM2* in BRCA cell division, they indicate that the PBK/TOPK-*GPSM2* pathway may be a promising molecular target for treating BRCA. In some other tumors, *GPSM2* phosphorylation was reduced and thus may be acted as an oncogenic agent. However, due to the limited current research reports, we cannot conclude the specific mechanism, but a complex cellular molecular mechanism is undoubtedly involved.

Gene mutation has been considered an important genetic cause of cancer. Although an average number of 3-6 mutations is thought to promote tumorigenesis, in most solid tumors, the total number of nonsynonymous mutations predicted to alter gene activity ranges from 40 to 100, and in some tumors (e.g., lung cancer), the number of mutations is as high as several hundred (Vogelstein et al., 2013). We found that the tumors with the highest *GPSM2* “Mutation” frequency (>6%) were UCEC. To our knowledge, no studies on *GPSM2* in UCEC have been reported. Nevertheless, the mechanism of development of UCEC, the most common gynecologic malignancy in the country, is related to tumor mutation load (Zhao et al., 2021). We found that in UCEC tumors, *GPSM2* mutation leads to reduced OS, PFS, DSS and DFS in patients, which may be associated with R17C mutation. This elucidates the impact of *GPSM2* on tumor prognosis at the genetic level, and inhibition of the *GPSM2* gene R17C mutation might be effective in suppressing UCEC disease progression, which is only speculation and hypothesis at present.

*GPSM2* plays a crucial role in establishing and maintaining cell polarity by determining the direction of spindle movement during mitosis (Deng et al., 2020a). Studies have shown that *GPSM2* expression decreases CD4 T+ cells in rheumatoid arthritis patients and can act as a promoter of regular T cell migration in healthy individuals (Dang et al., 2021a; Meyer et al., 2021). In addition, *GPSM2* can affect the infiltration of immune cells in the tumor microenvironment and promote tumor cell migration. Zhou et al. (Zhou et al., 2021) found that *GPSM2* can influence the level of immune cell infiltration and promote PAAD cell migration. Targeting *GPSM2* and its downstream genes may prolong PAAD patient survival time. Therefore, we explored the relationship between *GPSM2* expression and cancer-infiltrating immune cells.

Studies have found that cancer-associated fibroblasts play a role in cancer progression by contributing to extracellular matrix deposition and remodeling, EMT, invasion, metastasis, and therapy resistance (Asif et al., 2021). Many patients with advanced cancer have neutrophilia, and neutrophils recruited to tumors can acquire pro- or antitumor functions. In addition, tumor-associated neutrophils display functional plasticity (Shaul and Fridlender, 2018). In addition, tumor endothelial cells release and promote tumor progression by “vascular secretory factors.” Within the vasculature, tumor cells physically contact endothelial cells and interact with them through the juxtaposition of secretory and paracrine signals (Maishi and Hida, 2017).

We studied the relationship between *GPSM2* expression and the three immune cell types above. The results showed that *GPSM2* expression was positively correlated with cancer-associated fibroblast infiltration in PRAD and negatively correlated with BRCA. There was a positive correlation between *GPSM2* expression and neutrophils in BLCA, and *GPSM2* expression was negatively associated with endothelial cell infiltration in BRCA and STAD. The results showed that the correlation was different due to different tumor types. Immune cell infiltration is considered an essential factor in tumor development, and our findings are complementary to the report that *GPSM2* affects tumor progression through resistant action.

We performed pathway GO|KEGG pathway enrichment analysis of similar genes to explore the specific mechanism of *GPSM2* in cancer. The results showed that the enriched pathways were mainly “mitotic nuclear division,” “chromosome segmentation,” and “spindle,” which is consistent with previous reports. Previously, *GPSM2* showed a unique subcellular localization in mitosis; it localizes at the spindle cell periphery in metaphase, moves to the midzone in anaphase and is then concentrated at the midbody in telophase and during cytokinesis (Fukukawa et al., 2010). It is well known that cell polarization (Mohapatra et al., 2021) and cell cycle regulation (Dang et al., 2021b) are important factors contributing to tumorigenesis, which further validates our speculation.

In conclusion, our findings demonstrate the important value of *GPSM2* as a potential cancer marker. It is not only a potential prognostic biomarker but also a potential therapeutic target for specific types of cancer (e.g., PAAD) by affecting tumorigenesis-related pathways, but this requires substantial clinical validation.

## Conclusion

In summary, our comprehensive pan-cancer analysis of *GPSM2* revealed an association between *GPSM2* expression and clinical prognosis, protein phosphorylation, immune cell infiltration, tumor mutation burden, and microsatellite instability in human cancers. However, the small sample size and the lack of basic experimental validation are the limitations of this study. The study sample should be expanded in the future to ensure its reliability and further investigate the specific mechanisms between *GPSM2* and cancers by clinical samples.

## Data Availability

The original contributions presented in the study are included in the article/supplementary material, further inquiries can be directed to the corresponding author.

## Glossary

ACC: Adrenocortical carcinoma
ANLN: Anillin
BLCA: Bladder urothelial carcinoma
BRCA: Breast cancer
CAN: Copy number alteration
cBioPortal: cBio cancer genomics portal
CESC: Ceramic square cell carcinoma
CHOL: Cholangiocarcinoma
CKAP2 L: CKAP2 like
CKAP2: Cytoskeleton-associated protein 2
COAD: Colon adenocarcinoma
CPTAC: Clinical proteomic tumor analysis consortium
DCLRE1B: DNA cross-link repair 1B
DFS: Disease-free survival
DLBC: Diffuse large B-cell lymphoma
EMT: Epithelial-mesenchymal transition
ESCA: Esophageal carcinoma
FC: Fold change
GBM: Glioblastoma multiform
GEPIA2: Gene expression profiling interactive analysis 2
GO: Gene Ontology
GPSM2: G-protein signaling modulator 2
GTEx: Genotype-tissue expression
HNSC: Head and neck squamous cell carcinoma
HPA: Human protein atlas
KCTD5: Potassium channel tetramerization domain-5
KEGG: Kyoto encyclopedia of genes and genomes
KICH: Kidney chromophobe
KIF4A: Kinesin family member 4A
KIRC: Kidney renal clear cell carcinoma
KIRP: Kidney renal papillary cell carcinoma
LAML: Acute myeloid leukemia
LGG: Brain lower grade glioma
LGN: Leu-Gly-Asn repeat-enriched protein
LIHC: Liver hepatocellular carcinoma
LUAD: Lung adenocarcinoma
LUSC: Lung squamous cell carcinoma
MESO: Mesothelioma
OS: Overall survival
OV: Ovarian serous cystadenocarcinoma
PAAD: Pancreatic adenocarcinoma
PCPG: Pheochromocytoma and paraganglioma
PFS: Progression-free survival
PPI: Protein-protein interaction
PRAD: Prostate adenocarcinoma
RACGAP1: RAC GTPase activating protein 1
READ: Rectum adenocarcinoma
SARC: Sarcoma
SDS-PAGE: Sodium dodecyl sulfate-polyacrylamide gel electrophoresis
SKCM: Skin cutaneous melanoma
STAD: Stomach adenocarcinoma
TCGA: The Cancer Genome Atlas
TGCT: Testicular germ cell tumors
THCA: Thymic carcinoma: Thyroid carcinoma
THCA: Thymic carcinoma: Thyroid carcinoma
TPM: Transcripts per million
UCEC: Uterine corpus endometrial carcinoma
UCS: Uterine carcinosarcoma
UVM: Uveal melanoma
